# 4-Hy­droxy-3,5-dimeth­oxy-*N*-{4-[(5-methyl-1,2-oxazol-3-yl)sulfamo­yl]phen­yl}benzamide methanol monosolvate

**DOI:** 10.1107/S1600536811055991

**Published:** 2012-01-14

**Authors:** Wei-Gao Pan, Zhi-Dong Zhao, Peng Luo, Cui-Wu Lin, Jian-Hua Miao

**Affiliations:** a Guangxi Botanical Garden of Medicinal Plants, Nanning 530023, People’s Republic of China; b Guangxi University, College of Chemistry and Chemical Engineering, Nanning 530004, People’s Republic of China; c Guangxi Traditional Chinese Medicine University, Nanning 530001, People’s Republic of China

## Abstract

The title compound, C_19_H_19_N_3_O_7_S·CH_3_OH, was synthesized from syringic acid and sulfamethoxazole. The benzene rings make a dihedral angle of 41.8 (1)° and the isoxazole ring is twisted by 74.3 (1)° from the central benzene ring. The crystal packing features O—H⋯O and O—H⋯N hydrogen bonds in which the hy­droxy groups from the main mol­ecule and methanol solvent mol­ecules serve as donor groups.

## Related literature

For the biological activity of syringic acid and sulfamethoxazole, see: Wu *et al.* (2009 ▶); Itoh *et al.* (2009 ▶, 2010 ▶); Ramachandran & Raja (2010 ▶); Ma *et al.* (2007 ▶); Hida *et al.* (2005 ▶); Liu *et al.* (2003 ▶). For related structures, see: Camerman *et al.* (1979 ▶); Yan *et al.* (2009 ▶); Yasmeen *et al.* (2010 ▶).
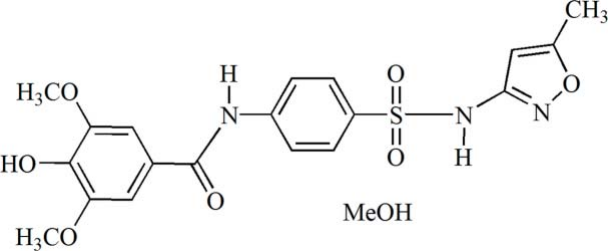


## Experimental

### 

#### Crystal data

C_19_H_19_N_3_O_7_S·CH_4_O
*M* *_r_* = 465.47
Monoclinic, 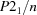

*a* = 12.133 (11) Å
*b* = 8.684 (8) Å
*c* = 20.983 (19) Å
β = 102.043 (13)°
*V* = 2162 (3) Å^3^
*Z* = 4
Mo *K*α radiation
μ = 0.20 mm^−1^
*T* = 296 K
0.35 × 0.24 × 0.20 mm

#### Data collection

Bruker SMART CCD area-detector diffractometer
Absorption correction: multi-scan (*SADABS*; Bruker, 2004 ▶) *T*_min_ = 0.933, *T*_max_ = 0.961
11585 measured reflections
3807 independent reflections
2900 reflections with *I* > 2σ(*I*)
*R*_int_ = 0.042

#### Refinement

*R*[*F* ^2^ > 2σ(*F* ^2^)] = 0.049
*wR*(*F* ^2^) = 0.140
*S* = 1.05
3807 reflections
298 parameters
H atoms treated by a mixture of independent and constrained refinement
Δρ_max_ = 0.34 e Å^−3^
Δρ_min_ = −0.48 e Å^−3^

### 

Data collection: *SMART* (Bruker, 2004 ▶); cell refinement: *SMART* (Bruker, 2004 ▶); data reduction: *SAINT*; program(s) used to solve structure: *SHELXS97* (Sheldrick, 2008 ▶); program(s) used to refine structure: *SHELXL97* (Sheldrick, 2008 ▶); molecular graphics: *SHELXTL* (Sheldrick, 2008 ▶); software used to prepare material for publication: *SHELXL97*.

## Supplementary Material

Crystal structure: contains datablock(s) I, global. DOI: 10.1107/S1600536811055991/bh2401sup1.cif

Structure factors: contains datablock(s) I. DOI: 10.1107/S1600536811055991/bh2401Isup2.hkl

Supplementary material file. DOI: 10.1107/S1600536811055991/bh2401Isup3.cml

Additional supplementary materials:  crystallographic information; 3D view; checkCIF report

## Figures and Tables

**Table 1:** Hydrogen-bond geometry (Å, °)

*D*—H⋯*A*	*D*—H	H⋯*A*	*D*⋯*A*	*D*—H⋯*A*
O2—H2⋯N3^i^	0.85 (3)	2.05 (3)	2.852 (4)	157 (3)
O8—H8⋯O2^ii^	0.94 (5)	2.12 (6)	3.004 (4)	156 (5)
O8—H8⋯O1^ii^	0.94 (5)	2.44 (5)	3.133 (4)	131 (4)

Symmetry codes: (i) 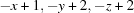; (ii) 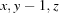.

**Fig. 1.**
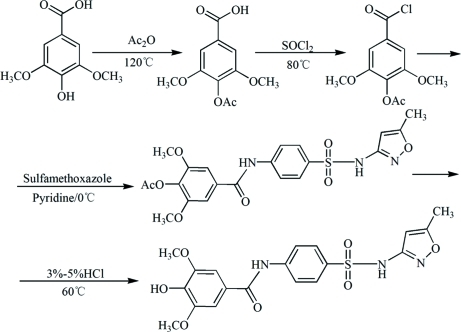
Synthesis of the title molecule.

**Fig. 2.**
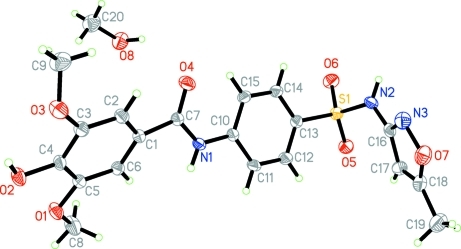
A view of the title compound, with 30% probability displacement ellipsoids.

**Fig. 3.**
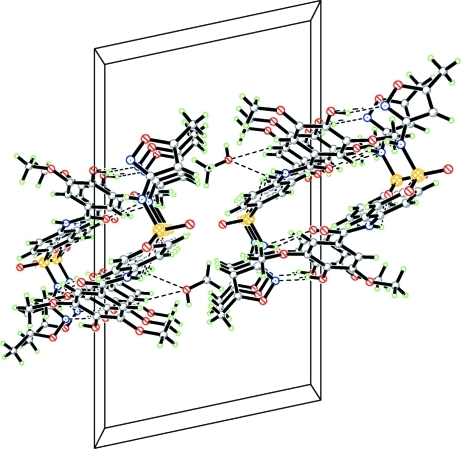
Crystal packing, viewed along the b axis, of the title complex. The O—H···O, O—H···N and N—H···O interactions are shown as dashed lines.

## supplementary crystallographic information

### Comment

Syringic acid, a natural compound occurring in many kinds of plant species, was synthesized and used widely in medicine, perfume, pesticide chemistry and organic synthetic industry. Syringic acid showed antifungal activity at high concentration (Wu *et al.*, 2009), anti-endotoxic effects (Liu *et al.*, 2003), hepatoprotective effect (Itoh *et al.*, 2009, 2010; Ramachandran & Raja, 2010). Sulfamethoxazole was usually used as anti-infective (anti-bacterial or anti-fungal) drug (Ma *et al.*, 2007; Hida *et al.*, 2005). Whether the title product (Fig. 1, 2 and 3) shows combined-effects (combining the activities of syringic acid with those of sulfamethoxazole) or has some novel properties should be investigated in future. Some structures closely related to the title compound were previously published, which include the syringic or sulfamethoxazole fragment (Camerman *et al.*, 1979; Yan *et al.*, 2009; Yasmeen *et al.*, 2010).

### Experimental

4000 mg (20 mmol) of syringic acid (4-hydroxy-3,5-dimethoxybenzoic acid) in 30 ml of acetic anhydride was stirred and refluxed at 120 °C for 2 h. 600 ml of water was added to the above solution to get precipitates (3610 mg) by method of pumping filtration. 3000 mg (12 mmol) of above dry precipitates in 20 ml of thionyl chloride was stirred and refluxed at 80 °C for 6 h under anhydrous conditions. The solution was removed under reduced pressure to get residue (3415 mg). 3100 mg (12 mmol) of the residue, 3040 mg (12 mmol) of sulfamethoxazole [4-amino-*N*-(5-methyl-3-isoxazolyl)benzenesulfonamide] and 6 ml of pyridine in 200 ml of THF were stirred at 0 °C for 2 h. and then at room temperature for 24 h. 4000 ml of water was added to the above reaction solution to get 4165 mg of precipitates after pumping filtration. 4000 mg (8.4 mmol) of these precipitates and 20 ml of 10 mmol.m*L*^-1^ hydrochloric acid in 150 ml of THF were stirred and refluxed at 60 °C for 1 h. 3000 ml of water was added to the hydrolytic solution to get product (2720 mg) after pumping filtration. This final synthetic product was detected by electrospray ionization mass spectroscopy (ESI) to give a molecular ion at *m*/*z* value of 432.0 ([M—H]^-^). This product was redissolved in mixed solution of THF and methanol and then left for evaporating at room temperature. After crystallization and recrystallization from the mixed solution, colorless crystals suitable for X-ray analysis were obtained (mp. 494–495 K).

### Refinement

H atoms bonded to C and N atoms were positioned geometrically with *d*(N—H) = 0.86 Å, *d*(C—H) = 0.93 (aromatic CH) or 0.96 Å (methyl CH_3_), and treated as riding atoms. Hydroxyl H atoms H2 and H8 were refined freely. For all H atoms, isotropic displacement parameters were calculated as *U*_iso_(H) = *xU*_eq_(N,*C*,*O*) with *x* = 1.2 or 1.5.

### Crystal data

**Table:**

C_19_H_19_N_3_O_7_S·CH_4_O	*F*(000) = 976
*M**_r_* = 465.47	*D*_x_ = 1.430 Mg m^−^^3^
Monoclinic, *P*2_1_/*n*	Melting point: 494 K
Hall symbol: -P 2yn	Mo *K*α radiation, λ = 0.71073 Å
*a* = 12.133 (11) Å	Cell parameters from 3807 reflections
*b* = 8.684 (8) Å	θ = 1.8–25.0°
*c* = 20.983 (19) Å	µ = 0.20 mm^−^^1^
β = 102.043 (13)°	*T* = 296 K
*V* = 2162 (3) Å^3^	Block, colourless
*Z* = 4	0.35 × 0.24 × 0.20 mm

### Data collection

**Table:**

Bruker SMART CCD area-detector diffractometer	3807 independent reflections
Radiation source: fine-focus sealed tube	2900 reflections with *I* > 2σ(*I*)
graphite	*R*_int_ = 0.042
φ and ω scans	θ_max_ = 25.0°, θ_min_ = 1.8°
Absorption correction: multi-scan (*SADABS*; Bruker, 2004)	*h* = −13→14
*T*_min_ = 0.933, *T*_max_ = 0.961	*k* = −10→10
11585 measured reflections	*l* = −23→24

### Refinement

**Table:**

Refinement on *F*^2^	Primary atom site location: structure-invariant direct methods
Least-squares matrix: full	Secondary atom site location: difference Fourier map
*R*[*F*^2^ > 2σ(*F*^2^)] = 0.049	Hydrogen site location: inferred from neighbouring sites
*wR*(*F*^2^) = 0.140	H atoms treated by a mixture of independent and constrained refinement
*S* = 1.05	*w* = 1/[σ^2^(*F*_o_^2^) + (0.0122*P*)^2^ + 1.2353*P*] where *P* = (*F*_o_^2^ + 2*F*_c_^2^)/3
3807 reflections	(Δ/σ)_max_ = 0.001
298 parameters	Δρ_max_ = 0.34 e Å^−^^3^
0 restraints	Δρ_min_ = −0.48 e Å^−^^3^
0 constraints	

### Fractional atomic coordinates and isotropic or equivalent isotropic displacement parameters (Å^2^)

**Table:**

	*x*	*y*	*z*	*U*_iso_*/*U*_eq_	
S1	0.18631 (5)	0.23253 (8)	1.00904 (3)	0.0372 (2)	
O1	0.29542 (15)	1.3250 (2)	0.77380 (10)	0.0480 (5)	
O2	0.49358 (17)	1.4549 (2)	0.80363 (10)	0.0463 (5)	
O3	0.66996 (15)	1.3222 (2)	0.88701 (11)	0.0533 (6)	
O4	0.51958 (15)	0.7933 (2)	0.92734 (11)	0.0539 (6)	
O5	0.06867 (14)	0.2401 (2)	1.00843 (10)	0.0463 (5)	
O6	0.23161 (17)	0.1065 (2)	0.97917 (10)	0.0501 (5)	
O7	0.24567 (19)	0.4522 (3)	1.22145 (11)	0.0599 (6)	
O8	0.40341 (19)	0.4397 (3)	0.65913 (12)	0.0639 (7)	
N1	0.33231 (17)	0.8292 (2)	0.91667 (11)	0.0384 (5)	
N2	0.25363 (17)	0.2263 (3)	1.08544 (11)	0.0398 (6)	
N3	0.3029 (2)	0.3712 (3)	1.17963 (13)	0.0522 (7)	
C1	0.4473 (2)	1.0346 (3)	0.88707 (12)	0.0331 (6)	
C2	0.5519 (2)	1.1046 (3)	0.90221 (13)	0.0373 (6)	
C3	0.5688 (2)	1.2450 (3)	0.87506 (13)	0.0375 (6)	
C4	0.4811 (2)	1.3181 (3)	0.83263 (13)	0.0343 (6)	
C5	0.3754 (2)	1.2467 (3)	0.81737 (13)	0.0358 (6)	
C6	0.3588 (2)	1.1071 (3)	0.84468 (13)	0.0351 (6)	
C7	0.4365 (2)	0.8765 (3)	0.91227 (13)	0.0339 (6)	
C8	0.1907 (2)	1.2484 (4)	0.75269 (19)	0.0664 (10)	
C9	0.7670 (2)	1.2299 (4)	0.88784 (19)	0.0639 (10)	
C10	0.3002 (2)	0.6841 (3)	0.93632 (13)	0.0344 (6)	
C11	0.1948 (2)	0.6720 (3)	0.95179 (14)	0.0420 (7)	
C12	0.1587 (2)	0.5344 (3)	0.97264 (14)	0.0436 (7)	
C13	0.2275 (2)	0.4053 (3)	0.97739 (13)	0.0353 (6)	
C14	0.3311 (2)	0.4152 (3)	0.96069 (14)	0.0395 (6)	
C15	0.3678 (2)	0.5532 (3)	0.94009 (14)	0.0405 (7)	
C16	0.2235 (2)	0.3183 (3)	1.13335 (13)	0.0371 (6)	
C17	0.1158 (2)	0.3594 (3)	1.14200 (15)	0.0462 (7)	
C18	0.1341 (3)	0.4427 (4)	1.19678 (15)	0.0505 (8)	
C19	0.0590 (3)	0.5249 (5)	1.23277 (19)	0.0758 (11)	
C20	0.4962 (3)	0.5218 (5)	0.64565 (19)	0.0711 (10)	
H1A	0.2792	0.8958	0.9062	0.046*	
H2	0.560 (3)	1.487 (4)	0.8163 (16)	0.060 (10)*	
H2B	0.3095	0.1639	1.0962	0.048*	
H2C	0.6109	1.0569	0.9308	0.045*	
H6A	0.2881	1.0608	0.8349	0.042*	
H8	0.418 (4)	0.420 (6)	0.704 (3)	0.15 (2)*	
H8A	0.1408	1.3128	0.7224	0.100*	
H8B	0.1578	1.2274	0.7896	0.100*	
H8C	0.2028	1.1534	0.7318	0.100*	
H9A	0.8330	1.2939	0.8963	0.096*	
H9B	0.7607	1.1802	0.8464	0.096*	
H9C	0.7729	1.1534	0.9214	0.096*	
H11A	0.1481	0.7579	0.9480	0.050*	
H12A	0.0884	0.5278	0.9835	0.052*	
H14A	0.3766	0.3284	0.9633	0.047*	
H15A	0.4378	0.5590	0.9287	0.049*	
H17A	0.0468	0.3343	1.1154	0.055*	
H19A	0.1035	0.5718	1.2711	0.114*	
H19B	0.0072	0.4531	1.2452	0.114*	
H19C	0.0178	0.6030	1.2053	0.114*	
H20A	0.4828	0.6303	0.6482	0.107*	
H20B	0.5060	0.4965	0.6026	0.107*	
H20C	0.5630	0.4944	0.6769	0.107*	

### Atomic displacement parameters (Å^2^)

**Table:**

	*U*^11^	*U*^22^	*U*^33^	*U*^12^	*U*^13^	*U*^23^
S1	0.0342 (4)	0.0333 (4)	0.0451 (4)	−0.0035 (3)	0.0105 (3)	0.0010 (3)
O1	0.0361 (10)	0.0457 (12)	0.0569 (13)	−0.0021 (8)	−0.0024 (9)	0.0198 (10)
O2	0.0402 (11)	0.0386 (12)	0.0569 (13)	−0.0080 (9)	0.0026 (9)	0.0146 (10)
O3	0.0371 (10)	0.0407 (12)	0.0760 (15)	−0.0075 (9)	−0.0018 (10)	0.0086 (11)
O4	0.0326 (10)	0.0447 (12)	0.0844 (16)	0.0043 (9)	0.0120 (10)	0.0239 (11)
O5	0.0321 (10)	0.0499 (13)	0.0566 (13)	−0.0079 (8)	0.0088 (9)	0.0033 (10)
O6	0.0560 (12)	0.0343 (11)	0.0639 (14)	−0.0033 (9)	0.0216 (10)	−0.0088 (10)
O7	0.0644 (14)	0.0680 (16)	0.0474 (13)	−0.0105 (11)	0.0120 (10)	−0.0069 (11)
O8	0.0547 (13)	0.0796 (18)	0.0555 (15)	−0.0104 (12)	0.0071 (11)	−0.0017 (13)
N1	0.0337 (11)	0.0314 (12)	0.0523 (14)	0.0070 (9)	0.0139 (10)	0.0123 (11)
N2	0.0346 (11)	0.0360 (13)	0.0485 (14)	0.0070 (9)	0.0080 (10)	0.0091 (11)
N3	0.0459 (14)	0.0581 (17)	0.0515 (16)	−0.0072 (12)	0.0080 (12)	0.0012 (13)
C1	0.0348 (13)	0.0315 (14)	0.0338 (14)	0.0018 (10)	0.0090 (11)	0.0035 (11)
C2	0.0366 (14)	0.0367 (16)	0.0374 (15)	0.0023 (11)	0.0051 (11)	0.0043 (12)
C3	0.0331 (13)	0.0337 (16)	0.0446 (16)	−0.0044 (11)	0.0057 (11)	0.0012 (12)
C4	0.0390 (14)	0.0283 (14)	0.0362 (14)	−0.0019 (10)	0.0094 (11)	0.0052 (12)
C5	0.0348 (14)	0.0369 (16)	0.0355 (15)	0.0049 (11)	0.0071 (11)	0.0060 (12)
C6	0.0323 (13)	0.0334 (15)	0.0401 (15)	−0.0014 (10)	0.0085 (11)	0.0036 (12)
C7	0.0325 (13)	0.0328 (15)	0.0371 (15)	0.0008 (10)	0.0085 (11)	0.0044 (12)
C8	0.0364 (16)	0.068 (2)	0.086 (3)	−0.0071 (15)	−0.0075 (16)	0.031 (2)
C9	0.0386 (16)	0.069 (2)	0.083 (3)	−0.0101 (15)	0.0116 (16)	−0.004 (2)
C10	0.0341 (13)	0.0337 (15)	0.0360 (14)	0.0015 (11)	0.0084 (11)	0.0064 (12)
C11	0.0307 (13)	0.0395 (16)	0.0573 (18)	0.0095 (11)	0.0125 (12)	0.0133 (14)
C12	0.0294 (13)	0.0449 (18)	0.0584 (19)	0.0023 (11)	0.0135 (12)	0.0104 (14)
C13	0.0342 (13)	0.0365 (15)	0.0356 (15)	−0.0004 (11)	0.0083 (11)	0.0017 (12)
C14	0.0392 (14)	0.0325 (15)	0.0504 (17)	0.0048 (11)	0.0180 (12)	0.0051 (13)
C15	0.0369 (14)	0.0353 (16)	0.0546 (18)	0.0033 (11)	0.0213 (12)	0.0048 (13)
C16	0.0369 (14)	0.0330 (15)	0.0413 (16)	−0.0035 (11)	0.0078 (12)	0.0120 (12)
C17	0.0379 (15)	0.0511 (19)	0.0500 (18)	0.0008 (12)	0.0104 (13)	0.0015 (15)
C18	0.0560 (19)	0.052 (2)	0.0468 (18)	−0.0015 (14)	0.0180 (15)	0.0083 (15)
C19	0.095 (3)	0.073 (3)	0.070 (3)	0.008 (2)	0.040 (2)	−0.002 (2)
C20	0.061 (2)	0.073 (3)	0.080 (3)	−0.0076 (18)	0.0143 (19)	−0.003 (2)

### Geometric parameters (Å, °)

**Table:**

S1—O5	1.426 (2)	C7—C1	1.486 (4)
S1—O6	1.427 (2)	C8—H8C	0.9600
S1—N2	1.641 (3)	C8—H8B	0.9600
S1—C13	1.755 (3)	C8—H8A	0.9600
O1—C5	1.367 (3)	C9—H9C	0.9600
O1—C8	1.420 (4)	C9—H9B	0.9600
O2—C4	1.357 (3)	C9—H9A	0.9600
O2—H2	0.85 (3)	C10—C15	1.394 (4)
O3—C3	1.374 (3)	C10—C11	1.388 (4)
O3—C9	1.421 (4)	C11—H11A	0.9300
O4—C7	1.227 (3)	C11—C12	1.375 (4)
O7—C18	1.347 (4)	C12—H12A	0.9300
O7—N3	1.415 (3)	C13—C12	1.389 (4)
O8—C20	1.410 (4)	C13—C14	1.376 (4)
O8—H8	0.94 (5)	C14—H14A	0.9300
N1—C7	1.350 (3)	C14—C15	1.379 (4)
N1—C10	1.406 (3)	C15—H15A	0.9300
N1—H1A	0.8600	C16—C17	1.403 (4)
N2—C16	1.392 (4)	C16—N3	1.301 (4)
N2—H2B	0.8600	C17—H17A	0.9300
C2—H2C	0.9300	C17—C18	1.337 (4)
C2—C1	1.383 (4)	C18—C19	1.482 (4)
C2—C3	1.379 (4)	C19—H19C	0.9600
C4—C5	1.400 (4)	C19—H19B	0.9600
C4—C3	1.391 (4)	C19—H19A	0.9600
C6—H6A	0.9300	C20—H20C	0.9600
C6—C1	1.394 (4)	C20—H20B	0.9600
C6—C5	1.373 (4)	C20—H20A	0.9600
			
S1—N2—H2B	118.8	C7—N1—C10	127.9 (2)
O1—C8—H8A	109.5	C10—C11—H11A	119.7
O1—C8—H8B	109.5	C10—C15—H15A	120.0
O1—C5—C6	124.9 (2)	C10—N1—H1A	116.1
O1—C5—C4	114.9 (2)	C11—C12—H12A	120.1
O1—C8—H8C	109.5	C11—C12—C13	119.9 (2)
O2—C4—C3	123.0 (2)	C11—C10—N1	117.5 (2)
O2—C4—C5	117.9 (2)	C11—C10—C15	119.0 (2)
O3—C9—H9A	109.5	C12—C11—H11A	119.7
O3—C9—H9B	109.5	C12—C11—C10	120.7 (2)
O3—C9—H9C	109.5	C12—C13—S1	120.2 (2)
O3—C3—C2	124.1 (2)	C13—C12—H12A	120.1
O3—C3—C4	115.4 (2)	C13—C14—H14A	119.8
O4—C7—N1	122.2 (2)	C13—C14—C15	120.4 (2)
O4—C7—C1	120.4 (2)	C14—C15—H15A	120.0
O5—S1—O6	120.54 (12)	C14—C15—C10	120.1 (2)
O5—S1—N2	107.67 (12)	C14—C13—S1	119.8 (2)
O6—S1—N2	104.17 (13)	C14—C13—C12	119.9 (2)
O5—S1—C13	108.68 (12)	C15—C10—N1	123.5 (2)
O6—S1—C13	108.85 (14)	C15—C14—H14A	119.8
O7—C18—C19	116.7 (3)	C16—N3—O7	104.8 (2)
O8—C20—H20A	109.5	C16—C17—H17A	127.6
O8—C20—H20B	109.5	C16—N2—H2B	118.8
O8—C20—H20C	109.5	C16—N2—S1	122.30 (18)
N1—C7—C1	117.3 (2)	C17—C18—C19	133.6 (3)
N2—S1—C13	105.97 (12)	C17—C18—O7	109.7 (3)
N2—C16—C17	129.2 (3)	C18—C19—H19C	109.5
N3—C16—N2	118.5 (2)	C18—C19—H19B	109.5
N3—C16—C17	112.2 (3)	C18—C19—H19A	109.5
C1—C2—H2C	119.9	C18—C17—H17A	127.6
C1—C6—H6A	119.8	C18—C17—C16	104.9 (3)
C2—C3—C4	120.5 (2)	C18—O7—N3	108.4 (2)
C2—C1—C7	118.0 (2)	C20—O8—H8	108 (3)
C2—C1—C6	119.7 (2)	H8A—C8—H8C	109.5
C3—C2—H2C	119.9	H8A—C8—H8B	109.5
C3—C2—C1	120.2 (2)	H8B—C8—H8C	109.5
C3—O3—C9	115.7 (2)	H9A—C9—H9C	109.5
C3—C4—C5	119.1 (2)	H9A—C9—H9B	109.5
C4—O2—H2	110 (2)	H9B—C9—H9C	109.5
C5—C6—H6A	119.8	H19A—C19—H19C	109.5
C5—C6—C1	120.3 (2)	H19A—C19—H19B	109.5
C5—O1—C8	116.1 (2)	H19B—C19—H19C	109.5
C6—C5—C4	120.2 (2)	H20A—C20—H20C	109.5
C6—C1—C7	122.0 (2)	H20A—C20—H20B	109.5
C7—N1—H1A	116.1	H20B—C20—H20C	109.5
			
S1—C13—C12—C11	175.8 (2)	C1—C6—C5—C4	−0.9 (4)
S1—C13—C14—C15	−175.4 (2)	C1—C6—C5—O1	177.9 (2)
S1—N2—C16—C17	39.3 (4)	C3—C4—C5—C6	0.7 (4)
S1—N2—C16—N3	−144.2 (2)	C3—C4—C5—O1	−178.1 (2)
O2—C4—C3—C2	−178.8 (2)	C3—C2—C1—C7	173.9 (2)
O2—C4—C3—O3	1.2 (4)	C3—C2—C1—C6	−0.6 (4)
O2—C4—C5—C6	179.1 (2)	C5—C4—C3—C2	−0.5 (4)
O2—C4—C5—O1	0.2 (4)	C5—C4—C3—O3	179.5 (2)
O4—C7—C1—C6	151.8 (3)	C5—C6—C1—C7	−173.5 (2)
O4—C7—C1—C2	−22.6 (4)	C5—C6—C1—C2	0.8 (4)
O5—S1—C13—C12	16.6 (3)	C7—N1—C10—C15	−15.7 (4)
O5—S1—C13—C14	−167.0 (2)	C7—N1—C10—C11	165.2 (3)
O5—S1—N2—C16	−42.3 (2)	C8—O1—C5—C4	174.2 (3)
O6—S1—C13—C12	149.6 (2)	C8—O1—C5—C6	−4.6 (4)
O6—S1—C13—C14	−34.0 (3)	C9—O3—C3—C4	−137.6 (3)
O6—S1—N2—C16	−171.4 (2)	C9—O3—C3—C2	42.4 (4)
N1—C10—C11—C12	−178.7 (3)	C10—C11—C12—C13	−1.0 (4)
N1—C10—C15—C14	179.1 (3)	C10—N1—C7—C1	177.2 (2)
N1—C7—C1—C6	−27.2 (4)	C10—N1—C7—O4	−1.8 (4)
N1—C7—C1—C2	158.4 (2)	C11—C10—C15—C14	−1.8 (4)
N2—C16—N3—O7	−177.1 (2)	C12—C13—C14—C15	0.9 (4)
N2—C16—C17—C18	177.1 (3)	C13—C14—C15—C10	0.3 (4)
N2—S1—C13—C12	−98.8 (2)	C13—S1—N2—C16	73.8 (2)
N2—S1—C13—C14	77.5 (2)	C14—C13—C12—C11	−0.6 (4)
N3—O7—C18—C17	0.6 (3)	C15—C10—C11—C12	2.1 (4)
N3—C16—C17—C18	0.4 (3)	C16—C17—C18—C19	177.9 (3)
N3—O7—C18—C19	−178.2 (3)	C16—C17—C18—O7	−0.6 (3)
C1—C2—C3—C4	0.5 (4)	C17—C16—N3—O7	−0.1 (3)
C1—C2—C3—O3	−179.5 (3)	C18—O7—N3—C16	−0.3 (3)

### Hydrogen-bond geometry (Å, °)

**Table:**

*D*—H···*A*	*D*—H	H···*A*	*D*···*A*	*D*—H···*A*
O2—H2···N3^i^	0.85 (3)	2.05 (3)	2.852 (4)	157 (3)
O8—H8···O2^ii^	0.94 (5)	2.12 (6)	3.004 (4)	156 (5)
O8—H8···O1^ii^	0.94 (5)	2.44 (5)	3.133 (4)	131 (4)

Symmetry codes: (i) −*x*+1, −*y*+2, −*z*+2; (ii) *x*, *y*−1, *z*.

## References

[bb1] Bruker (2004). *SMART*, *SAINT* and *SADABS* Bruker AXS Inc., Madison, Wisconsin, USA.

[bb2] Camerman, N., Chan, L. Y. Y., Yeung, H. W. & Mak, T. C. W. (1979). *Acta Cryst.* B**35**, 3004–3007.

[bb3] Hida, S., Yoshida, M., Nakabayashi, I., Miura, N. N., Adachi, Y. & Ohno, N. (2005). *Biol. Pharm. Bull.* **28**, 773–778. 10.1248/bpb.28.773 15863877

[bb4] Itoh, A., Isoda, K., Kondoh, M., Kawase, M., Kobayashi, M., Tamesada, M. & Yagi, K. (2009). *Biol. Pharm. Bull.* **32**, 1215–1219. 10.1248/bpb.32.1215 19571388

[bb5] Itoh, A., Isoda, K., Kondoh, M., Kawase, M., Watari, A., Kobayashi, M., Tamesada, M. & Yagi, K. (2010). *Biol. Pharm. Bull.* **33**, 983–987. 10.1248/bpb.33.983 20522963

[bb6] Liu, Y.-H., Fang, J.-G., Lei, T., Wang, W.-Q. & Lin, A.-H. (2003). *J. Huazhong Univ. Sci. Technol. Med. Sci.* **23**, 206–208. 10.1007/BF02859960 12973953

[bb7] Ma, M.-L., Cheng, Y.-Y., Xu, Z.-H., Xu, P., Qu, H.-O., Fang, Y.-J., Xu, T.-W. & Wen, L.-P. (2007). *Eur. J. Med. Chem.* **42**, 93–98. 10.1016/j.ejmech.2006.07.015 17095123

[bb8] Ramachandran, V. & Raja, B. (2010). *J. Basic Clin. Physiol. Pharmacol.* **21**, 369–385. 10.1515/jbcpp.2010.21.4.369 21305852

[bb9] Sheldrick, G. M. (2008). *Acta Cryst.* A**64**, 112–122. 10.1107/S0108767307043930 18156677

[bb10] Wu, H.-S., Luo, J., Liu, Y.-X., Chen, A.-Q., Tang, Z., Cao, Y., Chen, G., Mao, Z.-S., Huang, Q.-W. & Shen, Q.-R. (2009). *J. Eukaryot. Microbiol.* **56**, 386–387. 10.1111/j.1550-7408.2009.00417.x 19602085

[bb11] Yan, Y.-X., Hu, X.-D., Chen, J.-C., Sun, Y., Zhang, X.-M., Qing, C. & Qiu, M.-H. (2009). *J. Nat. Prod.* **72**, 308–311. 10.1021/np800719h 19133780

[bb12] Yasmeen, S., Murtaza, S., Akkurt, M., Khan, I. U. & Sharif, S. (2010). *Acta Cryst.* E**66**, o2264. 10.1107/S1600536810031132 PMC3007873 21588623

